# Sexual Dysfunction Following Breast Cancer Chemotherapy: A Cross-Sectional Study in Yogyakarta, Indonesia

**DOI:** 10.7759/cureus.41744

**Published:** 2023-07-11

**Authors:** Irfan Haris, Susanna H Hutajulu, Yufi K Astari, Juan A Wiranata, Irianiwati Widodo, Johan Kurnianda, Kartika W Taroeno-Hariadi, Mardiah S Hardianti, Ibnu Purwanto, Yayi S Prabandari

**Affiliations:** 1 Department of Internal Medicine, Faculty of Medicine, Public Health and Nursing, Universitas Gadjah Mada, Yogyakarta, IDN; 2 Division of Hematology and Medical Oncology, Department of Internal Medicine, Faculty of Medicine, Public Health and Nursing, Universitas Gadjah Mada/Dr Sardjito General Hospital, Yogyakarta, IDN; 3 Division of Hematology and Medical Oncology, Department of Internal Medicine, Dr. Sardjito General Hospital, Yogyakarta, IDN; 4 Department of Clinical Medicine, Faculty of Medicine, Public Health and Nursing, Universitas Gadjah Mada, Yogyakarta, IDN; 5 Department of Anatomical Pathology, Faculty of Medicine, Public Health and Nursing, Universitas Gadjah Mada/Dr. Sardjito General Hospital, Yogyakarta, IDN; 6 Division of Hematology and Medical Oncology, Department of Internal Medicine, Faculty of Medicine, Public Health and Nursing, Universitas Gadjah Mada/Dr. Sardjito General Hospital, Yogyakarta, IDN; 7 Department of Health Behavior, Environment, and Social Medicine, Faculty of Medicine, Public Health and Nursing, Universitas Gadjah Mada, Yogyakarta, IDN

**Keywords:** bmi, indonesia, sexual dysfunction, chemotherapy-related toxicity, breast cancer

## Abstract

Introduction

Sexual dysfunction is rarely studied in Indonesian patients with breast cancer. We aimed to assess the prevalence of sexual dysfunction symptoms following chemotherapy, as well as the pattern and the associated factors.

Methods

This cross-sectional study included 135 female breast cancer patients receiving primary chemotherapy. The present study measured the prevalence of sexual dysfunction symptoms using an e-questionnaire containing Common Toxicity Criteria for Adverse Events (CTCAE) version 4 at different time points. Other data included sociodemography, clinicopathology, treatment, and other concurrent symptom characteristics. Bivariate and multivariate logistic regression tests were used to analyze any association among variables.

Results

In the whole panel, 86 (63.7%) of 135 cases experienced sexual dysfunction. The most common symptom was vaginal dryness (45.9%), followed by decreased libido (45.2%), dyspareunia (13.3%), delayed orgasm (11.1%), and anorgasmia (8.9%). When observed at five different time points, the frequency of symptoms increased during chemotherapy and persisted until six months after completing treatment. Chemotherapy duration of >120 days was associated with a higher probability of vaginal dryness (p=0.012) and decreased libido (p=0.033). Spouse age ≥55 years old and body mass index (BMI) ≥23 kg/m^2^ were associated with a reduced probability of decreased libido (p=0.033 and 0.025, respectively). The presence of comorbidity was associated with a reduced probability of delayed orgasm (p=0.034).

Conclusions

A significant proportion of patients with breast cancer had sexual dysfunction following chemotherapy. Vaginal dryness, decreased libido, and dyspareunia were the commonest symptoms observed. Duration of chemotherapy, spouse age, BMI, and comorbidity were associated with the risk of sexual dysfunction occurrence.

## Introduction

Globocan 2018 data show that breast cancer is the second most common cancer worldwide, with an incidence of 2,088,849 per year after lung cancer. In women, breast cancer is the cancer with the highest incidence. It is number 4 in terms of cancer-related mortality, with 626,679 deaths per year [1]. In Indonesia, among all types of cancer, breast cancer has the highest cancer incidence with 58,256 new cases per year or 30.9% of all cancer for women and 16.7% of all cancer for both sexes. It is also a cancer with the second highest mortality with 22,692 deaths per year [2].

The increasing number of long-term breast cancer survivors has raised significant awareness on treatment-related adverse events, with particular attention to quality of life [3]. Female sexual dysfunction is an aspect of quality of life that has a high reported prevalence in the international literature (45-84%) [4-10]. Common symptoms include diminished vaginal lubrication, pain during intercourse (dyspareunia), decreased sense of arousal, and difficulty achieving orgasm [11,12]. Despite its high occurrence, sexual dysfunction in patients with cancer remains an under-explored issue in both Western (including the United Kingdom and the United States) and Eastern countries (including Iran and Malaysia) [4], and also expected in Indonesia.

Multiple studies have observed influencing factors of sexual dysfunction in patients undergoing cancer treatment. Older age [9,13], older spouse age [13], higher education [9,13], higher body mass index (BMI) [14], postmenopausal status [15], mastectomy [11], length of chemotherapy [16], and more advanced disease [13,16] were associated with significant reduction in sexual function. Other determinants are living arrangements [17] and molecular subtypes [13], although evidence is conflicting. Chemotherapy specifically can cause vaginal dryness, loss of sexual satisfaction, and loss of desire [9,13]. Anthracycline-based regimen is observed as a treatment-related regimen toward sexual dysfunction [16]. During their chemotherapy program, patients also often experience fatigue, anxiety, and depression, which further contribute to sexual dysfunction [12,16,18].

Only limited studies have reported the effect of breast cancer chemotherapy on sexual function in Indonesia [19,20], although breast cancer is the most common malignancy in the country [2] and chemotherapy remains a major treatment modality. Furthermore, none of these reported on cases in the researchers’ province of Yogyakarta, although the region has the highest cancer prevalence in Indonesia according to the National Health Survey [21]. The mentioned studies also have small sample sizes and have not investigated the sociodemographic and clinical factors that are associated with sexual dysfunction in effect estimations. Accordingly, this study aimed to identify the occurrence of sexual dysfunction symptoms following first-line chemotherapy for patients with breast cancer, as well as the pattern and the associated factors.

## Materials and methods

This is a cross-sectional study that included female patients with breast cancer who were recruited in a study aiming to analyze the risk of chemotherapy toxicities and determine their effect on survival. The main study started in July 2018 and planned to recruit a minimum of 200 cases. The registered patients visited and received their first chemotherapy treatment in the Hematology and Medical Oncology Division, “Tulip”/Integrated Cancer Clinic, Dr. Sardjito General Hospital, Yogyakarta, Indonesia. Women aged ≥18 years with histopathologically proven breast cancer without terminal condition and severe comorbidities were included. Cases received chemotherapy as neoadjuvant treatment (before surgery), adjuvant treatment (after surgery), or palliative treatment with or without surgery. The present study consecutively included patients who were registered in the main study between July 2018 and March 2021 (n=215). Patients were excluded if they were unmarried or singles, received less than three cycles of chemotherapy, had incomplete information, or were lost to follow-up. A total of 135 patients were included in the final analysis. Data collection from the main study was conducted between May and October 2021.

Sociodemographic data, clinicopathological data, and chemotherapy-related symptoms were obtained from the main study database. The parent study used an electronic/e-questionnaire for symptom assessment that was adopted for the Indonesian language from the Patient-Reported Outcomes version of the Common Toxicity Criteria for Adverse Events (PRO-CTCAE) version 4 [22]. Translation to Bahasa Indonesia was done by two general practitioners and then reviewed by a medical oncologist, a cardiologist, and a neurologist. A user-friendly and customizable questionnaire form was developed for editing of question items. Any symptom occurrence was listed and accompanied by descriptions of severity as described in the PRO-CTCAE. Every one and two weeks after each chemotherapy cycle, the patients were interviewed face-to-face by trained researchers at the ambulatory clinic. We continued to interview patients every one to six months after chemotherapy completion to identify any long-term chemotherapy effects. Due to the COVID-19 pandemic situation, some interviews were conducted through phone calls.

We collected data on chemotherapy-related sexual dysfunction symptoms as outcome variables. These consisted of vaginal dryness, decreased libido, dyspareunia, delayed orgasm, and anorgasmia. Independent variables included chemotherapy regimen (anthracycline-based vs other regimens) and chemotherapy duration (≤120 days vs >120 days). Data of sociodemographic data, clinicopathologic data, surgery type, and other concurrent chemotherapy-related symptoms were also included as potential confounders. The sociodemographic data consisted of age (<51 years vs ≥51 years, referred to the mean of patient age), education (high vs low education, referred to a cut-off of nine years of education based on the median from our patients), spouse age (≤55 years vs >55 years, referred to the mean of spouse age), and living arrangements (nuclear family, defined as couple-only or couple with children, vs extended family, defined as living with other family members, with or without a spouse). The clinicopathologic data consisted of BMI (<23 kg/m^2^ or underweight to normal vs ≥23 kg/m^2^ or overweight to obesity, referred to WHO BMI cut-off for Asian populations), menopausal status (post-menopause vs pre-menopause), comorbidity (yes vs no, referred to any comorbidity presence of hypertension, diabetes mellitus, dyslipidemia, or hyperuricemia), clinical stage based on the 7th edition American Joint Committee on Cancer (AJCC) staging system (early: stage I-II; locally advanced: stage III; and metastatic: stage IV), and molecular subtypes (luminal A, luminal B [either with human epidermal growth factor receptor 2, HER-2, negative or HER-2 positive], HER-2 enriched, and triple negative) using immunohistochemical study. Type of surgery was described as mastectomy and without mastectomy. Concurrent chemotherapy-induced symptoms included fatigue, anxiety, and depression.

Sexual dysfunction symptoms were retrieved in five particular points of time (T) to see the dynamic pattern of the symptoms. These included the first chemotherapy cycle (T0), after the third cycle (T1), one week after the last chemotherapy cycle (T2), one month after chemotherapy completion (T3), and six months (T4) after chemotherapy completion.

We presented characteristic data and the cumulative occurrence of sexual dysfunction and concurrent symptoms in any cycle of chemotherapy as frequency. Using McNemar tests, the frequency of sexual dysfunction symptoms was analyzed at the different time points. Bivariate logistic regression tests were used to analyze any association between variables and the frequency of sexual dysfunction symptoms, with 95% confidence interval (CI) and significance set as a p-value of <0.05. Logistic regression was not applicable to any interaction between variables with zero occurrences. All factors with a p-value of <0.25 in the bivariate analyses were further analyzed using multivariate logistic regression. We used SPSS version 22 (IBM Corp., Armonk, NY) for statistical analysis.

## Results

**Figure 1:**
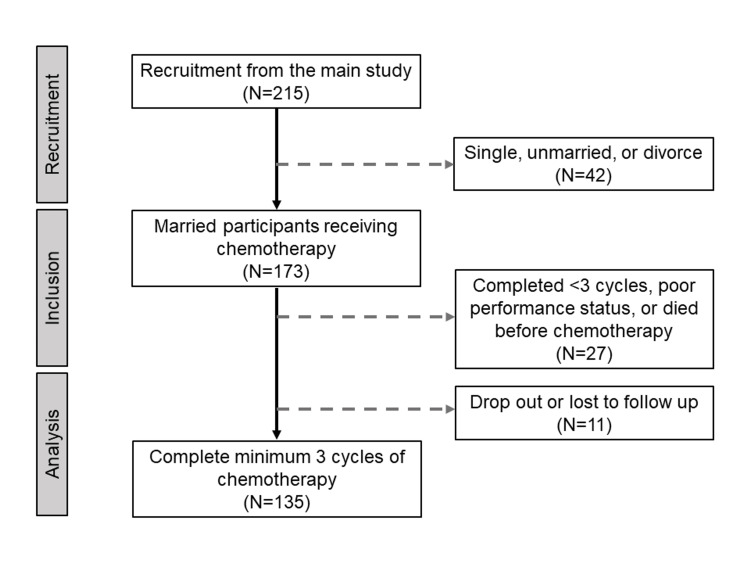
Flow diagram for the study's recruitment, inclusion, and analysis process.

The present study consecutively included 215 patients who were registered in the main study between July 2018 and March 2021. Patients were excluded because they were not married (n=42), received less than three cycles of chemotherapy with poor performance status or died before chemotherapy initiation (n=27), and had incomplete information or lost to follow-up (n=11). A total of 135 patients were included in the final analysis (Figure 1). 

Table 1 displays the baseline characteristics of all patients. Participants were predominately aged <51 years and having spouses aged <55 years. Most of the participants had high education level (57%) and lived with nuclear family (55.6%). Most of them had BMI ≥ 23 kg/m^2^ (60%), had at least one comorbidity (53.3%), had a postmenopausal status (57%), had a locally advanced stage (45.9%), had luminal-B subtype (39.3%), underwent mastectomy (83.7%), received anthracycline-based regimen (80.7%), and received chemotherapy for >120 days (65.9%). As many as 86 patients (63.7%) experienced one or more sexual dysfunction symptoms at least once in any given treatment cycle. The commonest symptoms were vaginal dryness (45.9%) and decreased libido (45.2%), followed by dyspareunia (13.3%), delayed orgasm (11.1%), and anorgasmia (8.9%). Almost all participants experienced fatigue (97.0%), and majority of cases had anxiety (73.3%) and depression (67.4%).

**Table 1:** Sociodemographic and clinical characteristics of the patients (n =135). SD, standard deviation; BMI, body mass index; HER-2, human epidermal growth factor receptor 2

Variables	Frequency
	n (%)
Age (mean ± SD)	50.34 ± 7.63
<51 years	70 (51.9)
≥51 years	65 (48.1)
Spouse age (mean ± SD; n=119)	53.58 ± 9.06
<55 years	64 (47.4)
≥55 years	55 (40.7)
Living arrangement (n=122)	
Nuclear family	75 (55.6)
Extended family	47 (34.8)
Education level	
Low education (<9 years)	58 (43)
High education (≥9 years)	77 (57)
BMI (mean ± SD)	24.19 ± 4.33
<23 kg/m^2^	54 (40)
≥23 kg/m^2^	81 (60)
Menopausal status	
Pre-menopause	58 (43)
Post-menopause	77 (57)
Comorbidities	
Without comorbidity	63 (46.7)
With comorbidity(-ies)	72 (53.3)
Stage	
Early breast cancer	44 (32.6)
Locally advanced breast cancer	62 (45.9)
Metastatic breast cancer	29 (21.5)
Molecular subtype	
Luminal A	35 (25.9)
Luminal B (HER-2 negative or positive)	53 (39.3)
HER-2 enriched	22 (16.3)
Triple negative	25 (18.5)
Mastectomy	
No	22 (16.3)
Yes	113 (83.7)
Anthracycline-based chemotherapy	
No	26 (19.3)
Yes	109 (80.7)
Chemotherapy duration (mean ± SD)	139.59 ± 35.22
≤120 days	46 (34.1)
>120 days	89 (65.9)

**Figure 2:**
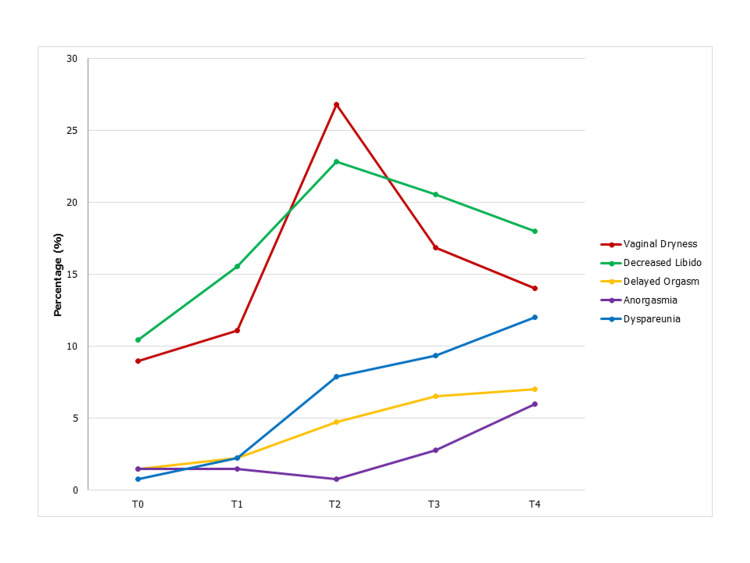
Female sexual dysfunction occurrence and pattern during chemotherapy and after chemotherapy completion. T0=after the first cycle of chemotherapy; T1=after the third cycle of chemotherapy; T2=after chemotherapy completion; T3=one month after chemotherapy completion; T4=six months after chemotherapy completion.

Table 2 shows the distribution and comparison of symptom frequency at the five different time points (T0-T4). At T1, all sexual dysfunction symptoms were slightly increased compared to T0. At T2, the occurrence of vaginal dryness and decreased libido dropped, and dyspareunia abruptly increased, while delayed orgasm only slightly increased and anorgasmia decreased. At T3, vaginal dryness and decreased libido started to decrease, while delayed orgasm, anorgasmia, and dyspareunia still increased. At T4, vaginal dryness and decreased libido decreased, anorgasmia and dyspareunia increased, and delayed orgasm persisted (Figure 2).

**Table 2:** Distribution and comparison of sexual dysfunction frequency at baseline and other time points during and after chemotherapy. T0=after the first cycle of chemotherapy, T1=after the third cycle of chemotherapy, T2=after chemotherapy completion, T3=one month after chemotherapy completion, T4=six months after chemotherapy completion *Asymptotic significance. #Exact significance.

	T0 (n =134)	T1 (n =135)	P-value	T2 (n =127)	P-value	T3 (n =107)	P-value	T4 (n =100)	P-value
	%	%		%		%		%	
Vaginal dryness									
Yes	9.0	11.1	0.607	26.8	<0.001*	16.8	0.115	14.0	0.359
No	91.0	88.9		73.2		83.2		86.0	
Decreased libido									
Yes	10.4	15.6	0.167	22.8	0.005*	20.4	0.021^#^	18.0	0.043^#^
No	89.6	84.4		77.2		79.4		82.0	
Delayed orgasm									
Yes	1.5	2.2	1.000	4.7	0.063	6.5	0.016^#^	7.0	0.070
No	98.5	97.8		95.3		93.5		93.0	
Anorgasmia									
Yes	1.5	1.5	1.000	0.8	1.000	2.8	0.625	6.0	0.125
No	98.5	98.5		99.2		97.2		94.0	
Dyspareunia									
Yes	0.7	2.2	0.500	7.9	0.004^#^	9.3	0.002^#^	12.0	<0.001^#^
No	99.3	97.8		92.1		90.7		88.0	

No significant differences in all symptom occurrences were found between T1 and T0 (p-values >0.05). At T2, the occurrence of vaginal dryness, decreased libido, and dyspareunia significantly increased compared to T0 (26.8% vs 9.0%, p 0.001; 22.8% vs 10.4%, p=0.005; and 7.9% vs 0.7%, p=0.004, respectively). At T3, decreased libido, delayed orgasm, and dyspareunia showed significant changes compared to T0 (20.6% vs 10.4%, p=0.021; 6.5% vs 1.5%, p=0.016; and 9.3% vs 0.7%, p=0.002, respectively). At T4, only decreased libido and dyspareunia showed a significant increase compared to T0 (18% vs 10.4%, p=0.043; and 12% vs 0.7%, p <0.001, respectively) (Table 2).

Table 3 shows the results of the logistic regression analyses of the variables that were associated with sexual dysfunction. In the multivariate analyses, chemotherapy duration >120 days was a significant determinant for a higher probability of vaginal dryness (OR 3.55, 95% CI 1.32-9.55, p=0.012) and decreased libido (OR 3.67, 95% CI 1.11-12.09, p=0.033). Spouse age ≥55 years old and BMI ≥23 kg/m^2^ were significantly associated with lower occurrence of decreased libido (OR 0.25, 95% CI 0.07-0.89, p=0.033; and OR 0.33, 95% CI 0.13-0.87, p=0.025, respectively). The presence of comorbidity was the only significant factor associated with decreased delayed orgasm (OR 0.23, 95% CI 0.06-0.89, p=0.034).

**Table 3:** Factors associated with female sexual dysfunction occurrence during chemotherapy program. *Bivariate logistic regression. **Multivariate logistic regression. HER-2, human epidermal growth factor receptor 2; NA, not applicable

	Vaginal dryness				Decreased libido				Delayed orgasm				Anorgasmia				Dyspareunia			
	OR (95% CI)*	P-value*	OR (95% CI)**	P-value**	OR (95% CI)*	P-value*	OR (95% CI)**	P-value**	OR (95% CI)*	P-value*	OR (95% CI)**	P-value**	OR (95% CI)*	P-value*	OR (95% CI)**	P-value**	OR (95% CI)*	P-value*	OR (95% CI)**	P-value**
Age																				
<51 years old	Ref				Ref		Ref		Ref		Ref		Ref				Ref			
≥51 years old	0.71 (0.36-1.40)	0.325			0.52 (0.26-1.04)	0.064	1.45 (0.39-5.32)	0.572	0.50 (0.16-1.55)	0.230	0.66 (0.18-2.49)	0.542	0.75 (0.23-2.49)	0.639			0.84 (0.31-2.28)	0.736		
Spouse age																				
<55 years old	Ref				Ref		Ref		Ref				Ref				Ref			
≥55 years old	0.68 (0.33-1.41)	0.303			0.42 (0.19-0.87)	0.021	0.25 (0.07-0.89)	0.033	1.02 (0.35-3.02)	0.970			0.55 (0.16-1.93)	0.350			0.79 (0.28-2.23)	0.653		
Living arrangement																				
Nuclear family	Ref				Ref		Ref		Ref				Ref				Ref			
Extended family	0.68 (0.33-1.43)	0.310			0.63 (0.29-1.31)	0.214	0.61 (0.25-1.52)	0.291	0.77 (0.25-2.42)	0.660			0.78 (0.22-2.75)	0.698			1.14 (0.40-3.23)	0.809		
Education level																				
Low	Ref		Ref		Ref				Ref		Ref		Ref				Ref			
High	1.77 (0.88-3.54)	0.107	1.76 (0.79-3.95)	0.159	1.31 (0.66-2.61)	0.441			2.25 (0.68-7.47)	0.185	1.74 (0.46-6.57)	0.415	1.57 (0.45-5.47)	0.483			1.21 (0.44-3.35)	0.708		
BMI																				
<23 kg/m^2^	Ref				Ref		Ref		Ref				Ref				Ref			
≥23 kg/m^2^	0.67 (0.34-1.34)	0.260			0.44 (0.22-0.88)	0.021	0.33 (0.13-0.87)	0.025	0.54 (0.19-1.60)	0.269			0.93 (0.28-3.09)	0.902			0.81 (0.29-2.20)	0.680		
Menopausal status																				
Not yet menopause	Ref		Ref		Ref				Ref				Ref				Ref			
Already menopause	0.59 (0.29-1.17)	0.129	0.55 (0.25-1.20)	0.133	0.71 (0.36-1.41)	0.330			0.63 (0.21-1.84)	0.393			1.06 (0.32-3.53)	0.924			0.93 (0.34-2.53)	0.892		
Comorbidities																				
Without comorbidity	Ref				Ref		Ref		Ref		Ref		Ref				Ref			
With comorbidity(-ies)	1.26 (0.64-2.49)	0.504			0.58 (0.29-1.15)	0.117	0.89 (0.36-2.22)	0.806	0.28 (0.08-0.92)	0.037	0.23 (0.06-0.89)	0.034	0.86 (0.26-2.83)	0.809			0.86 (0.32-2.31)	0.761		
Stage																				
Early breast cancer	Ref		Ref		Ref		Ref		Ref				Ref				Ref		Ref	
Locally advanced breast cancer	1.64 (0.75-3.59)	0.212	1.79 (0.73-4.41)	0.203	1.09 (0.51-2.37)	0.818	0.84 (0.30-2.29)	0.728	0.84 (0.24-2.93)	0.779			1.74 (0.42-7.14)	0.442			1.02 (0.35-2.92)	0.976	0.81 (0.25-2.65)	0.729
Metastatic breast cancer	0.88 (0.34-2.31)	0.799	0.70 (0.23-2.16)	0.537	0.49 (0.18-1.32)	0.159	0.27 (0.06-1.14)	0.075	1.25 (0.31-5.09)	0.758			1.01 (0.16-6.46)	0.990			1.19 (0.02-1.62)	0.129	0.16 (0.02-1.59)	0.164
Molecular subtype																				
Luminal A	Ref				Ref		Ref		Ref		Ref		Ref		Ref		Ref		Ref	
Luminal B	1.56 (0.66-3.69)	0.315			1.74 (0.73-4.12)	0.208	1.33 (0.41-4.29)	0.631	3.88 (0.79-18.71)	0.096	3.33 (0.60-18.42)	0.168	5.17 (0.61-44.05)	0.133	4.96 (0.56 43.93)	0.150	4.32 (0.89-20.86)	0.068	4.99 (0.92-27.25)	0.063
HER-2 enriched	1.25 (0.43-3.67)	0.685			0.76 (0.25-2.28)	0.627	0.56 (0.13-2.39)	0.433	1.65 (0.22-12.65)	0.630	1.98 (0.21-18.99)	0.554	3.40 (0.29-39.92)	0.330	3.67 (0.29-45.28)	0.311	1.65 (0.22-12.65)	0.630	1.71 (0.21-13.83)	0.613
Triple negative	1.18 (0.42-3.33)	0.757			0.63 (0.21-1.84)	0.395	0.27 (0.06-1.27)	0.096	0.69 (0.06-8.03)	0.765	0.67 (0.05-9.51)	0.767	2.96 (0.25-34.54)	0.387	6.28 (0.48-82.63)	0.162	2.25 (0.35-14.58)	0.395	2.71 (0.38-19.35)	0.321
Mastectomy																				
No	Ref				Ref				Ref				Ref				Ref		Ref	
Yes	1.60 (0.62-4.12)	0.328			0.64 (0.26-1.59)	0.337			0.75 (0.19-2.92)	0.681			NA	NA			3.72 (0.47-29.51)	0.214	3.78 (0.43-33.35)	0.231
Anthracycline-based																				
No	Ref		Ref		Ref		Ref		Ref		Ref		Ref				Ref			
Yes	2.21 (0.89-5.51)	0.089	0.95 (0.30-3.04)	0.937	2.13 (0.85-5.31)	0.105	0.44 (0.09-2.11)	0.302	1.63 (0.34-7.69)	0.540	6.82 (0.79-59.20)	0.081	2.81 (0.35-22.77)	0.334			1.22 (0.33-4.58)	0.765		
Chemotherapy duration																				
≤120 days	Ref		Ref		Ref		Ref		Ref				Ref		Ref		Ref		Ref	
>120 days	3.11 (1.45-6.69)	0.004	3.55 (1.32-9.55)	0.012	2.56 (1.20-5.43)	0.015	3.67 (1.11-12.09)	0.033	8.40 (1.07-66.05)	0.043			6.35 (0.79-50.78)	0.082	8.27 (0.91-75.39)	0.061	2.91 (0.79-10.61)	0.107	3.21 (0.81-12.75)	0.098
Fatigue																				
No	Ref				Ref				Ref				Ref				Ref			
Yes	NA	NA			0.82 (0.11-5.99)	0.845			0.36 (0.04-3.69)	0.389			NA	NA			NA	NA		
Anxiety																				
No	Ref		Ref		Ref		Ref		Ref		Ref		Ref		Ref		Ref			
Yes	3.46 (1.48-8.09)	0.004	2.78 (0.96-8.10)	0.061	2.76 (1.21-6.33)	0.016	1.75 (0.49-6.25)	0.390	5.77 (0.73-45.53)	0.097	3.58 (0.38-33.84)	0.266	0.47 (0.14-1.59)	0.227	0.32 (0.09-1.18)	0.088	1.96 (0.53-7.23)	0.310		
Depression																				
No	Ref		Ref		Ref		Ref		Ref		Ref		Ref				Ref			
Yes	2.39 (1.12-5.09)	0.024	1.39 (0.52-3.68)	0.514	2.29 (1.08-4.88)	0.032	1.80 (0.54-6.04)	0.340	3.50 (0.75-16.25)	0.110	2.09 (0.36-12.33)	0.412	1.50 (0.39-5.84)	0.559			1.82 (0.56-5.89)	0.319		

## Discussion

This is the first Indonesian study that captures a comprehensive portrait of the dysfunction sexual problem in a clinical setting. The high prevalence (63.7%) of sexual dysfunction among our local breast cancer patients confirmed previous reports from other parts of Indonesia (63.1-93.5%) [19,20,23] and other countries (45-84%) [4-10]. The sexual disturbances occurred regardless of the surgery treatment, as also seen by other studies [11,24,25]. Sexual dysfunction symptoms are generally due to chemotherapy-induced ovarian failure, leading to decreased libido and anthropic vaginitis, manifesting as vaginal dryness and dyspareunia. Similar to previous studies, the most common symptoms in our studies were vaginal dryness, decreased libido, and dyspareunia [11].

We found that vaginal dryness and decreased libido increased from the start until chemotherapy completion, indicating an association with cumulative treatment dose. Although improved after the end of chemotherapy, both symptoms did not return to baseline. Meanwhile, delayed orgasm, anorgasmia, and dyspareunia slowly increased during chemotherapy and continued till six months after the end of chemotherapy. Farthmann et al. found that sexual activity dropped from 71.9% before chemotherapy to 47% at the end of chemotherapy and increased again to 65.7% in six months after chemotherapy. A similar effect was seen in sexual pleasure, discomfort, and orgasm [25]. Harirchi et al. reported that more patients experienced sexual dysfunction in three months after treatment compared to pre-treatment (84% vs 52%) with higher decreased score of female sexual function index for desire and lubrication compared to other domains [10]. Several studies have reported a persistence or worsening of sexual dysfunction symptoms many years after treatment completion. One of the most common reasons is an irreversible ovarian damage by chemotherapy agents resulting in permanent menopause [12,24,26].

Our findings showed risk factors that are linked with the occurrence of certain sexual dysfunction symptoms. Patients with a spouse aged ≥55 years old had a lower risk for decreased libido. This observation differs from a study that found that women with a spouse aged ≥55 years had greater sexual dysfunction [13]. The discrepancy could be attributable to other factors that might influence sexual desire, such as emotional intimacy and relationship stability [6,12], which were not examined in our study. Furthermore, we reported a significant association between higher BMI and a lower likelihood of decreased libido. Baumgart et al. also showed that obesity was associated with less reporting of dyspareunia [18], although this did not support the findings of another study [14]. Having a greater amount of adipose tissue increased the aromatization of testosterone into estradiol [18]. Estradiol levels are perceived to have a substantial link to sexual desire and responsiveness [27]. Consistent with other results [11,28], the main sexual dysfunction problems in our study were related to lubrication and arousal. Beside the partner’s age and BMI, longer chemotherapy duration increased probability of vaginal dryness and decreased libido in our local patients, supporting previous observations [16]. Remarkably, the presence of comorbidity was associated with a reduced risk of delayed orgasm. A study indicated that individuals with more illness possibly have lower expectations and therefore relatively higher sexual satisfaction [29].

The high prevalence of sexual dysfunction in the present study urges an improved cancer care delivery. Sociocultural barriers in Indonesia may influence patient’s understanding about and response to the sexuality topic. Most Indonesian patients never actively report such problems to their physician, probably because they feel embarrassed to talk about this openly. This may lead to underdiagnosis and undermanagement of this emotionally devastating issue. Time constraints for specialized consultations and prejudices about patient interests concerning talking about this topic are among the reasons on the doctor side [8,30]. Assessment of sexual dysfunction needs to be incorporated in the routine care and should be considered as essential as their clinical cancer management in order to treat them holistically. Education about these treatment-related problems needs to be imparted prior to cancer treatment. This proactive approach will allow patients to be more mentally prepared. Furthermore, they may openly discuss with their clinicians whenever the symptoms appear so that they can be referred to an expert who can help with proper consultation and treatment.

Inclusion from the existing cohort study may introduce some limitations. Firstly, the study collected chemotherapy-related toxicity data after the first cycle of treatment. As a consequence, the present study did not have information about pre-existing sexual dysfunction symptoms before chemotherapy started (before T0). Nonetheless, the increased symptom occurrence during chemotherapy program indicated a natural and temporal relationship between sexual dysfunction and chemotherapy. Secondly, some patients underwent other treatment modalities after chemotherapy, such as radiation and/or hormonal therapy. These approaches may influence our interpretation of sexual problems at T4. Irrespective of these limitations, this study is the first to picture dysfunction sexual symptoms of Indonesian cancer patients with complex content. Further studies are needed to address the mentioned limitations and provide a better understanding of the problem.

## Conclusions

A significant proportion of local patients with breast cancer experienced sexual dysfunction during and after chemotherapy completion. Vaginal dryness, decreased libido, and dyspareunia were the commonest symptoms found. Duration of chemotherapy, spouse age, BMI, and the presence of comorbidity were factors associated with sexual dysfunction symptoms. These factors may serve as tools to identify patients who are at risk of developing sexual dysfunction and should be assessed before, during, and after finishing cancer treatment.
